# The Yin–Yang of Stress and Senescence: Integrated Stress Response and SASP Crosstalk in Stem Cell Fate, Regeneration, and Disease

**DOI:** 10.32604/biocell.2025.072273

**Published:** 2026-01-23

**Authors:** Douglas M. Ruden

**Affiliations:** Department of Obstetrics and Gynecology, C. S. Mott Center for Human Growth and Development, Institute of Environmental Health Sciences, Wayne State University, Detroit, MI 48201, USA

**Keywords:** Integrated stress response (ISR), senescence-associated secretory phenotype (SASP), stem cells, activating transcription factor 4 (ATF4), eukaryotic initiation factor 2 alpha (eIF2α), inflammation, cancer

## Abstract

Stem cell fate decisions are increasingly understood through the dynamic interplay of two fundamental stress-adaptive programs: the integrated stress response (ISR) and the senescence-associated secretory phenotype (SASP). These pathways act as a Yin–Yang system, balancing beneficial and detrimental outcomes across development, tissue homeostasis, and disease. On the yin (protective) side, transient ISR activation and acute SASP signaling foster adaptation, embryonic patterning, wound healing, and regeneration. On the yang (maladaptive) side, chronic ISR signaling and unresolved SASP output drive stem cell exhaustion, fibrosis, inflammation, and tumorigenesis. This duality highlights their roles as both guardians and disruptors of stem cell integrity. Mechanistically, ISR regulates translational control via eukaryotic initiation factor 2 alpha (eIF2α) phosphorylation and activating transcription factor 4 (ATF4)-dependent transcription, while SASP reprograms the extracellular milieu through cytokines, growth factors, and proteases. Their crosstalk creates feedback loops that shape tissue niches and long-term stem cell potential. Framing ISR–SASP interactions through a Yin–Yang lens underscores the balance between resilience and decline, to offer new insights into regenerative medicine, anti-aging interventions, and cancer therapeutics.

## 1 Introduction

**Figure 1:**
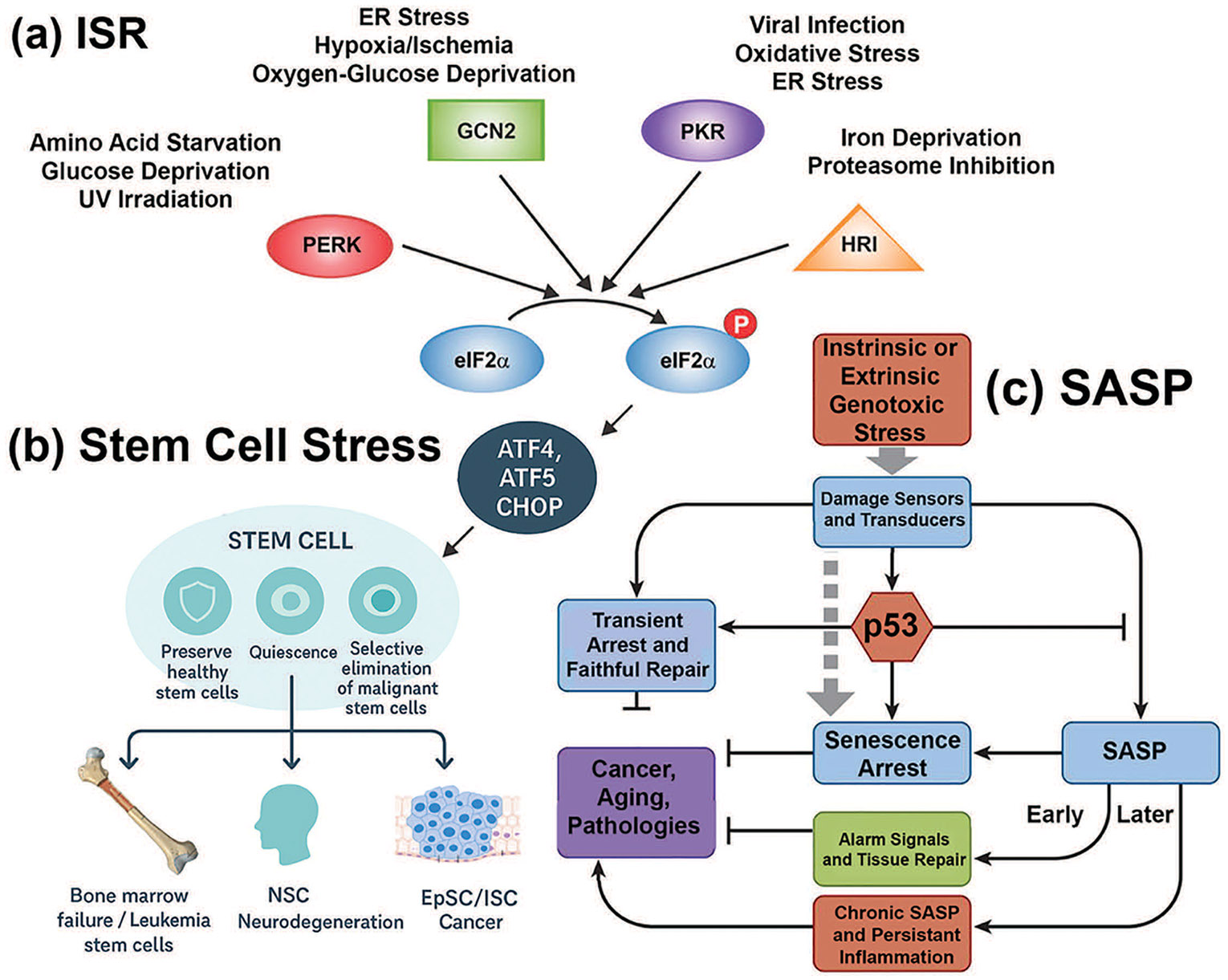
The integrated stress response (ISR) and senescence-associated secretory phenotype (SASP) in stem cell fate decisions. (**a**) ISR pathway. The four principal stress-sensing kinases—PERK (ER stress), GCN2 (amino acid deprivation), PKR (viral dsRNA), and HRI (oxidative stress/heme depletion)—converge to phosphorylate eIF2α, leading to global translational repression but selective translation of ATF4, ATF5, and CHOP. These transcription factors regulate adaptive or apoptotic programs that determine stem cell survival and lineage fate. (**b**) Stem cell stress outcomes. ISR activity influences diverse stem cell compartments, including hematopoietic stem cells (HSCs), neural stem cells (NSCs), and epithelial stem cells (epidermal/intestinal). Protective outcomes are represented by a shield icon, denoting preservation of healthy stem cells and regenerative potential. Detrimental outcomes are represented by a target icon, indicating selective elimination of malignant or senescent cells. Disease contexts associated with each compartment are illustrated: bone marrow failure and leukemia stem cells (HSCs), neurodegeneration and repair (NSCs), and epithelial regeneration or cancer (epithelial stem cells). (**c**) SASP pathway. Genotoxic stress—arising intrinsically within the cell or extrinsically from the microenvironment—activates DNA damage sensors that signal through p53. Early responses include cell cycle arrest and transient SASP suppression, promoting senescent arrest and acute alarm signaling. Over time, unresolved stress drives the full SASP program, characterized by chronic secretion of cytokines, growth factors, and proteases. This late-stage SASP fuels pathological outcomes that parallel the stem cell fates in (**b**), including bone marrow failure, neurodegeneration, and tumorigenesis. Together, panels (**a**–**c**) illustrate how ISR and SASP form a coupled Yin–Yang regulatory system that guides stem cell adaptation versus degeneration across development, aging, and disease. Abbreviations: integrated stress response (ISR); senescence-associated secretory phenotype (SASP); eukaryotic initiation factor 2 alpha (eIF2α), Protein Kinase RNA-Like ER Kinase (PERK), General control nonderepressible 2 (GCN2), protein kinase R (PKR), heme-regulated inhibitor (HRI), activating transcription factor 4,5 (ATF4,5), C/EBP Homologous Protein (CHOP), neural stem cell (NSC), intestinal stem cell (ISC), epithelial stem cell (EpSC), p53 kinase (p53)

Cellular stress responses have evolved to maintain tissue homeostasis by enabling cells to sense, adapt, or undergo programmed elimination in the face of environmental and metabolic perturbations. Two central processes that exemplify this adaptive capacity are the integrated stress response (ISR) (Fig. 1a) [1,2] and the senescence-associated secretory phenotype (SASP) (Fig. 1c) [3]. Although traditionally studied in distinct contexts, these pathways are increasingly recognized as intersecting networks that shape the biology of stem cells in health and disease (Fig. 1b) [4].

The ISR is a highly conserved signaling axis that modulates protein synthesis in response to diverse stressors such as nutrient deprivation, hypoxia, oxidative stress, and endoplasmic reticulum (ER) stress (Fig. 1a) [1,2]. The pathway converges on phosphorylation of the eukaryotic initiation factor eukaryotic initiation factor 2 alpha (eIF2α), resulting in global translational attenuation coupled with selective upregulation of stress-responsive genes, particularly the activating transcription factor 4 (ATF4) [5]. Through this mechanism, the ISR functions as a rheostat that balances adaptive survival with apoptotic elimination [5]. In stem cells, where proteostasis and energy balance are tightly linked to fate decisions, ISR activation can preserve quiescence, promote lineage bias, or trigger differentiation depending on context [4].

In parallel, the SASP represents a hallmark feature of senescent cells, characterized by the secretion of pro-inflammatory cytokines, chemokines, growth factors, and matrix remodeling enzymes (Fig. 1c) [3]. Initially described as a driver of aging-related pathologies [5], the SASP is now recognized as a context-dependent mediator of tissue remodeling, regeneration, and developmental morphogenesis [6]. Transient, developmentally programmed senescence contributes to embryonic patterning [7], while injury-induced senescence accelerates wound healing and tissue repair [8]. Conversely, chronic or unresolved SASP signaling promotes fibrosis, inflammation, and tumorigenesis.

Stem cells are uniquely poised at the intersection of ISR and SASP biology (Fig. 1b) [9]. They require stress response pathways to sustain self-renewal and differentiation capacity, yet are vulnerable to maladaptive senescence signaling that can compromise their function [10]. Emerging evidence suggests that ISR activity in senescent cells directly shapes SASP composition, particularly by amplifying pro-inflammatory and proteolytic factors [11]. Conversely, SASP-mediated paracrine signaling can feed back onto stem cells, inducing plasticity or exhaustion depending on exposure dynamics [12].

The convergence of ISR and SASP thus represents a critical axis in developmental biology, tissue homeostasis, and age-associated disease [13]. This review synthesizes recent advances in understanding ISR–SASP crosstalk in stem cells, highlighting mechanisms, developmental roles, pathological consequences, and therapeutic opportunities. We also discuss methodological approaches that have enabled the dissection of these pathways, and we outline future directions aimed at leveraging ISR–SASP biology for regenerative and anti-aging interventions.

## 2 Methods

As this is a review article, Section 2 describes the systematic approach to literature selection, integration, and synthesis. We performed a structured literature search across PubMed, Web of Science, and Scopus databases from 2000–2025, using combinations of keywords including “integrated stress response,” “ISR,” “eIF2α phosphorylation,” “ATF4,” “senescence-associated secretory phenotype,” “SASP,” “stem cells,” “developmental senescence,” “inflammation,” and “regeneration.” We also screened preclinical trial databases and citation networks of key ISR–SASP papers to identify additional mechanistic or therapeutic studies.

Studies in mammalian models were prioritized, with supplemental evidence drawn from invertebrate systems where mechanistic insights were uniquely informative. Inclusion criteria required primary data on ISR or SASP signaling, stem cell outcomes, or disease relevance; exclusion criteria were studies lacking mechanistic detail, non-English publications, or conference abstracts without peer-reviewed follow-up.

Primary research articles, reviews, and preprints were included, with emphasis on mechanistic studies that directly interrogated ISR or SASP function in stem cells, development, or tissue repair. For ISR-related findings, we extracted details on eIF2α kinases (PERK, GCN2, PKR, HRI), downstream transcriptional programs, and functional consequences for stem cell fate. For SASP-related findings, we categorized secretome composition, temporal dynamics, and paracrine effects across developmental, regenerative, and pathological contexts. When available, we noted whether outcomes were acute/reversible or chronic/irreversible, to align with the proposed Yin–Yang model of ISR–SASP crosstalk.

The integration strategy was thematic, with findings organized into three domains: (1) ISR and stem cell biology, (2) SASP and development/regeneration, and (3) ISR–SASP crosstalk. Within each domain, evidence was synthesized into conceptual frameworks that highlight shared mechanisms, unique contexts, and therapeutic implications. All extracted studies were cataloged in an internal reference database, and key mechanistic examples are summarized in Table 1 to enhance transparency.

We also considered high-throughput multi-omics datasets, including transcriptomic, proteomic, and secretomic analyses, that provided system-level insights into ISR–SASP interactions. In cases where ISR and SASP were studied independently, we analyzed overlapping pathways, such as regulation of translation, NF-κB signaling, and cytokine production, to infer points of convergence. Therapeutic studies targeting ISR or SASP nodes were abstracted separately and synthesized into Table 2, with categorization by intervention point, disease context, and reported outcomes.

To ensure rigor, conflicting findings were examined considering experimental models, cell types, and stressors. For example, discrepancies in ISR’s effect on stem cell differentiation often reflected variations in nutrient stress versus ER stress. Similarly, SASP’s regenerative versus deleterious effects were parsed by distinguishing transient versus chronic senescence models. Where disagreements remained unresolved, we explicitly note these as knowledge gaps in the Discussion.

This methodology allows for a balanced synthesis of mechanistic insights, developmental roles, and translational opportunities, while acknowledging limitations such as model-specific effects, incomplete temporal mapping of ISR–SASP interactions, and a lack of longitudinal studies in human systems. Future reviews may benefit from meta-analytical approaches once sufficient quantitative datasets accumulate across ISR and SASP research.

## 3 Discussion

### 3.1 The Yin–Yang Model of ISR–SASP Crosstalk

**Figure 2:**
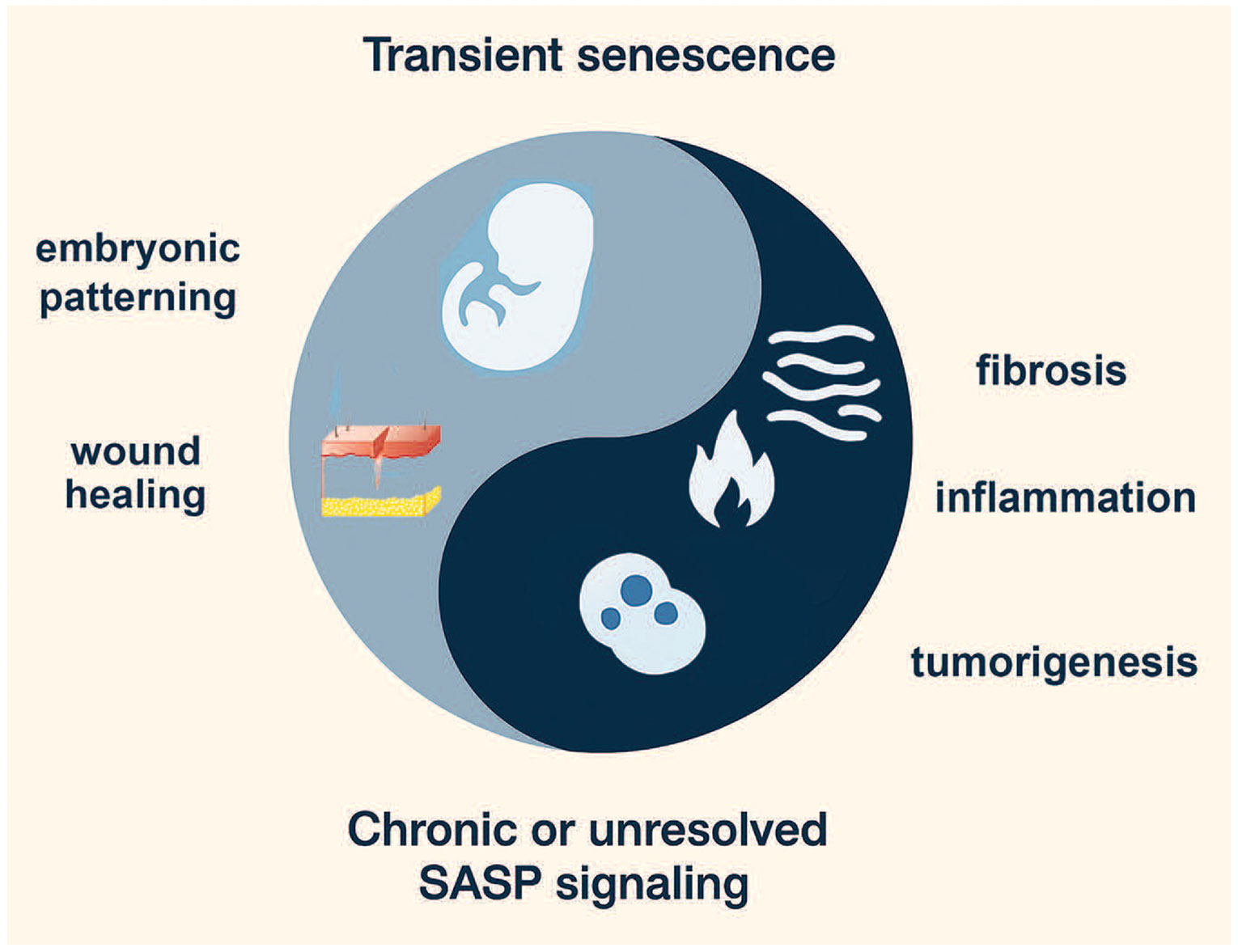
Yin–Yang model of senescence functions in development, regeneration, and disease. The figure illustrates the dual nature of senescence-associated signaling. The yin (light blue) represents transient, developmentally programmed, or injury-induced senescence, which contributes to embryonic patterning and accelerates wound healing and tissue repair. In contrast, the yang (dark blue) reflects chronic or unresolved senescence-associated secretory phenotype (SASP) signaling, which drives pathological outcomes such as fibrosis, chronic inflammation, and tumorigenesis. Together, this balance highlights the context-dependent roles of senescence in shaping stem cell biology, tissue homeostasis, and disease. The figure was made with ChatGPT5 and Photoshop

We propose a “Yin–Yang” model to conceptualize how the integrated stress response (ISR) and the senescence-associated secretory phenotype (SASP) jointly regulate stem cell fate decisions (Fig. 2; Table 1). In this framework, protective (“Yin”) and maladaptive (“Yang”) outcomes are not fixed categories but dynamic states along a continuum. Three criteria define this balance. First, temporal dynamics: acute and transient activation of ISR or SASP can be adaptive, allowing cells to restore proteostasis, repair damage, or signal for regeneration, whereas chronic and sustained activation often tips toward dysfunction, senescence, or degeneration [32]. Second, intensity of signaling: mild or moderate stress can bias stem cells toward survival, quiescence, or lineage choice, while overwhelming stress activates irreversible checkpoints such as apoptosis or senescence [32,33]. Third, functional outcomes: ISR–SASP interactions produce context-dependent effects, ranging from tissue renewal and developmental patterning [6] to chronic inflammation, stem cell exhaustion, or oncogenic transformation [34,35].

Importantly, the Yin–Yang model emphasizes a continuum rather than a binary switch, where protective and pathological outcomes arise from the same signaling modules depending on timing, strength, and cellular context. For example, ISR-mediated translational control can either preserve hematopoietic stem cell quiescence under nutrient stress or, if prolonged, drive maladaptive senescence programs [36,37]. Similarly, the SASP can promote tissue repair in acute settings but, when chronic, foster inflammatory microenvironments that impair stem cell function and promote cancer [6,31]. Recognizing this spectrum is essential for interpreting ISR–SASP crosstalk in development, aging, and disease, and for designing interventions that tilt the balance toward adaptive states [31].

### 3.2 ISR as a Guardian of Stem Cell Integrity

The integrated stress response (ISR) acts as a central quality-control mechanism for stem cells, balancing adaptation with the preservation of regenerative potential (Fig. 1a and Table 1) [4,15]. At the molecular level, ISR signaling converges on eukaryotic initiation factor 2 alpha (eIF2α) phosphorylation, which transiently suppresses global protein synthesis while permitting selective translation of stress-adaptive transcripts, particularly those governed by ATF4 [5,38]. In hematopoietic stem cells (HSCs), this pathway preserves quiescence during nutrient limitation and oxidative stress, ensuring a reserve of stem cells is maintained for future regenerative needs [4,14,39].

Similarly, epidermal stem cells rely on ISR activity during amino acid deprivation, biasing differentiation programs toward epidermal fates while restraining hair follicle entry [15,16]. In neural stem cells, ISR signaling mitigates ER stress and maintains proteostasis, safeguarding against premature differentiation [17,18]. By dynamically adjusting protein synthesis, metabolism, and survival, the ISR provides a stress-buffering shield: cells capable of adapting resume renewal and tissue contribution, while cells with insurmountable damage undergo apoptosis or senescence [4,40]. This selective mechanism maintains the long-term integrity of stem cell compartments, ensuring tissue resilience throughout life.

### 3.3 SASP as a Double-Edged Regulator of Stem Cell Fate

The senescence-associated secretory phenotype (SASP) represents the paracrine counterpart to the cell-intrinsic ISR, together forming a Yin–Yang system of stem cell regulation (Fig. 1c) [12]. Whereas ISR primarily dictates the survival-versus-death balance within stressed stem cells [4], the SASP reshapes the microenvironment, influencing both neighboring stem cells and tissue repair dynamics [12].

SASP initiation begins with intrinsic or extrinsic genotoxic stress that activates canonical DNA damage sensors, leading to stabilization of p53 and p21-mediated cell cycle arrest [41,42]. Early SASP signaling is often transient and context-dependent, functioning as an acute “alarm” to mobilize immune clearance and stimulate regenerative pathways (Table 1) [43,44]. For example, short-lived SASP activity in epithelial stem cell niches facilitates wound healing and tissue remodeling, echoing a yin state of controlled stress adaptation [45].

Over time, unresolved damage drives a chronic SASP, orchestrated largely through NF-κB and C/EBPβ transcriptional programs [46,47]. This prolonged pro-inflammatory secretome shifts the balance toward a yang state, fostering fibrosis, chronic inflammation, and tumor promotion (Fig. 1b,c) [48]. In hematopoietic stem cells, chronic SASP signaling contributes to bone marrow failure and the expansion of pre-leukemic clones [49]; in neural stem cells, it exacerbates neurodegenerative processes by impairing neurogenesis and promoting inflammatory gliosis [50,51]; in epithelial compartments, it reinforces malignant transformation through persistent cytokine and growth factor release [3,31].

Recent evidence highlights mechanistic crosstalk between ISR and SASP. For instance, ATF4-driven metabolic rewiring in stressed stem cells modulates SASP factor production, while chronic SASP cytokines (e.g., IL-6, IL-8) can feed back to activate ISR via PERK and PKR signaling in recipient cells [4,6,52]. This bidirectional loop links intrinsic stress-buffering with extrinsic niche remodeling, tightly integrating cellular and tissue-level outcomes [4,6,52]. Importantly, this interaction occurs along a continuum: acute SASP provides transient regenerative cues, whereas chronic SASP drives degeneration, with mixed or partial SASP states occupying the intermediate spectrum [6,41,53].

Thus, the SASP operates as a double-edged regulator of stem cell fate—capable of mobilizing repair when tightly controlled, but also fueling pathology when persistent. Explicitly positioning the SASP within the Yin–Yang framework clarifies how temporal dynamics, stress intensity, and tissue context determine whether SASP signaling promotes resilience or degeneration.

### 3.4 Wnt Signaling as a Central Axis of the Differentiation-Associated Stress Response

The Wnt signaling network functions as a developmental axis that couples environmental stress with lineage specification and tissue repair (Table 1) [22,23]. Canonical Wnt/β-catenin signaling broadly maintains stemness and self-renewal in multiple stem cell populations [54], including embryonic stem cells [55] and adult intestinal crypt progenitors [54,56]. Under homeostatic conditions, canonical Wnt activity stabilizes β-catenin, enabling the transcription of renewal-associated targets that preserve tissue growth and regenerative capacity [57].

Stress, however, destabilizes this equilibrium and can bias Wnt signaling toward non-canonical branches. Non-canonical Wnt pathways, including planar cell polarity and calcium-dependent cascades, are activated under redox, metabolic, or ischemic stress [58]. This switch functions as a developmental checkpoint within the development associated stress response (DASR): it restrains indefinite self-renewal and promotes context-dependent differentiation or tissue remodeling. For example, ischemic injury in muscle and heart progenitors activates Wnt5a-mediated non-canonical signaling, which promotes repair but also depletes the long-term stem cell reservoir [59].

In embryogenesis, the canonical-to-non-canonical Wnt transition safeguards patterning fidelity under fluctuating nutrient and oxygen conditions [60,61]. By constraining unbalanced renewal, Wnt-mediated DASR ensures that differentiation trajectories proceed despite environmental instability. In adult tissues, however, maladaptive persistence of non-canonical Wnt activity has been linked to pathology: chronic Wnt5a-mediated non-canonical signaling contributes to fibrotic remodeling, impaired stem cell pool maintenance, and even tumor progression [62,63].

Thus, Wnt signaling is not a static determinant of stemness, but rather a stress-responsive rheostat within the DASR framework [64,65]. By toggling between canonical and non-canonical outputs, it integrates environmental inputs, developmental timing, and repair demands—determining whether stem cells preserve self-renewal, commit to differentiation, or exhaust their regenerative capacity [64,65].

The Wnt pathway indeed occupies a central position in our proposed Yin–Yang framework for integrating the integrated stress response (ISR) and the developmental adaptive stress response (DASR). However, Wnt signaling is not acting in isolation; rather, it operates within a network of feedback regulators and transcriptional hubs that modulate cell fate under stress. Among these, FoxO1 represents a particularly compelling candidate as an additional nodal regulator [66,67]. FoxO1 not only mediates stress-induced transcriptional programs downstream of AKT and AMPK but also interfaces directly with Wnt/β-catenin signaling [66,67]. Under stress, FoxO1 can sequester β-catenin away from TCF/LEF transcriptional complexes, shifting the balance from proliferation and differentiation (DASR-like) toward cytoprotective and quiescent states (ISR-like) [66,67]. Thus, FoxO1 may serve as a molecular switch that dynamically tunes Wnt output depending on energetic and redox context [66,67]. This crosstalk supports the notion that the Yin–Yang relationship between ISR and DASR is governed by a small number of multifunctional signaling hubs, of which Wnt and FoxO1 are central exemplars.

### 3.5 Developmental Stress as Yin: Patterning and Wound Healing

The “yin” (protective) side of the developmental stress response reflects its constructive role in patterning, morphogenesis, and wound repair (Fig. 2). During embryogenesis, controlled stress signaling sculpts lineage allocation and tissue organization [60,61]. For example, hypoxia-inducible factors (HIFs) activated by physiologic oxygen gradients orchestrate vascular and neural development, guiding pattern formation through spatially restricted stress cues [17,18]. Similarly, oxidative stress at low levels serves as a morphogen-like signal, regulating cardiac and skeletal muscle development [68,69].

Beyond embryogenesis, these stress-responsive pathways are redeployed in adult wound healing. Acute stress responses at injury sites activate ISR, Wnt, and TGF-β networks, which together coordinate cell migration, fibroblast activation, angiogenesis, and tissue closure [70]. In this context, stress is neither purely damaging nor protective but operates as a developmental toolkit that can be re-engaged in regenerative settings [70]. The yin perspective thus emphasizes stress as an instructive, organizing force that promotes adaptation and renewal [60,61].

### 3.6 Pathological Stress as Yang: Fibrosis, Inflammation, and Tumors

In contrast, the “yang” (maladaptive) dimension of developmental stress emerges when adaptive programs become chronic or dysregulated, fueling pathology (Fig. 2) [29-31]. Prolonged activation of ISR and Wnt pathways shifts their roles from protective to maladaptive, driving fibrotic scarring, chronic inflammation, and tumorigenesis [29-31]. In fibrotic disease, persistent TGF-β and stress-induced Wnt activation lead to sustained fibroblast activation and extracellular matrix deposition, replacing regenerative repair with rigid scarring [71]. Inflammation represents another maladaptive outcome, where chronic ISR and NF-κB activation perpetuate cytokine storms, exhausting tissue stem cell pools and disrupting regenerative balance [72].

Tumor biology can be seen as an extreme form of stress maladaptation: cancer stem cells exploit ISR to resist nutrient and oxidative stress, while hijacking Wnt and other developmental pathways to sustain unchecked proliferation [29-31]. The Yin–Yang model thus underscores the duality of stress responses—protective and patterning in acute, controlled contexts, but pathological and destructive when stress is excessive or unresolved. Understanding this continuum provides a framework for therapies that re-bias stress signaling toward adaptation and repair while preventing pathological drift.

### 3.7 Molecular Crosstalk between ISR and SASP

A growing body of evidence indicates that the integrated stress response (ISR) and the senescence-associated secretory phenotype (SASP) are linked not only conceptually but also through direct molecular cross-regulation [4,11]. At the core of the ISR is the phosphorylation of eIF2α by stress-sensing kinases (PERK, PKR, HRI, and GCN2), which reduces global protein synthesis while selectively enhancing translation of activating transcription factor 4 (ATF4) [2]. ATF4, in turn, activates transcriptional programs that overlap substantially with SASP regulators [2]. For instance, ATF4 can upregulate IL-6 and IL-8, two canonical SASP cytokines, through cooperation with NF-κB and AP-1 [3,41]. Conversely, chronic ISR signaling promotes expression of CHOP, which not only drives apoptosis but also contributes to the pro-inflammatory arm of SASP [2].

Reciprocally, SASP factors themselves can reinforce ISR signaling in recipient cells. Cytokines such as IFN-γ and TNF-α activate PKR, leading to renewed eIF2α phosphorylation [73,74], while amino acid–depleting enzymes secreted during senescence (e.g., indoleamine 2,3-dioxygenase) activate GCN2 [75,76], thereby amplifying ISR activity. This creates a feed-forward loop in which senescent cells impose stress on neighboring stem or progenitor cells, nudging them toward maladaptive fates [77,78]. In tissue contexts, this ISR–SASP crosstalk contributes to both regenerative responses—such as transient activation of ISR to facilitate wound healing [4,6]—and degenerative outcomes, including fibrosis and stem cell exhaustion under chronic stress [4,6].

Thus, the mechanistic interface between ISR and SASP can be conceptualized as a dynamic circuit: ATF4-driven ISR promotes SASP-like transcriptional outputs, while SASP-derived cytokines and metabolites reactivate ISR in surrounding cells [4,6]. Identifying the molecular nodes of this circuit, such as ATF4–NF-κB convergence or PKR-mediated ISR activation by SASP cytokines, will be critical for therapeutic targeting [6,79].

### 3.8 Therapeutic Modulation of ISR–SASP Crosstalk

**Figure 3:**
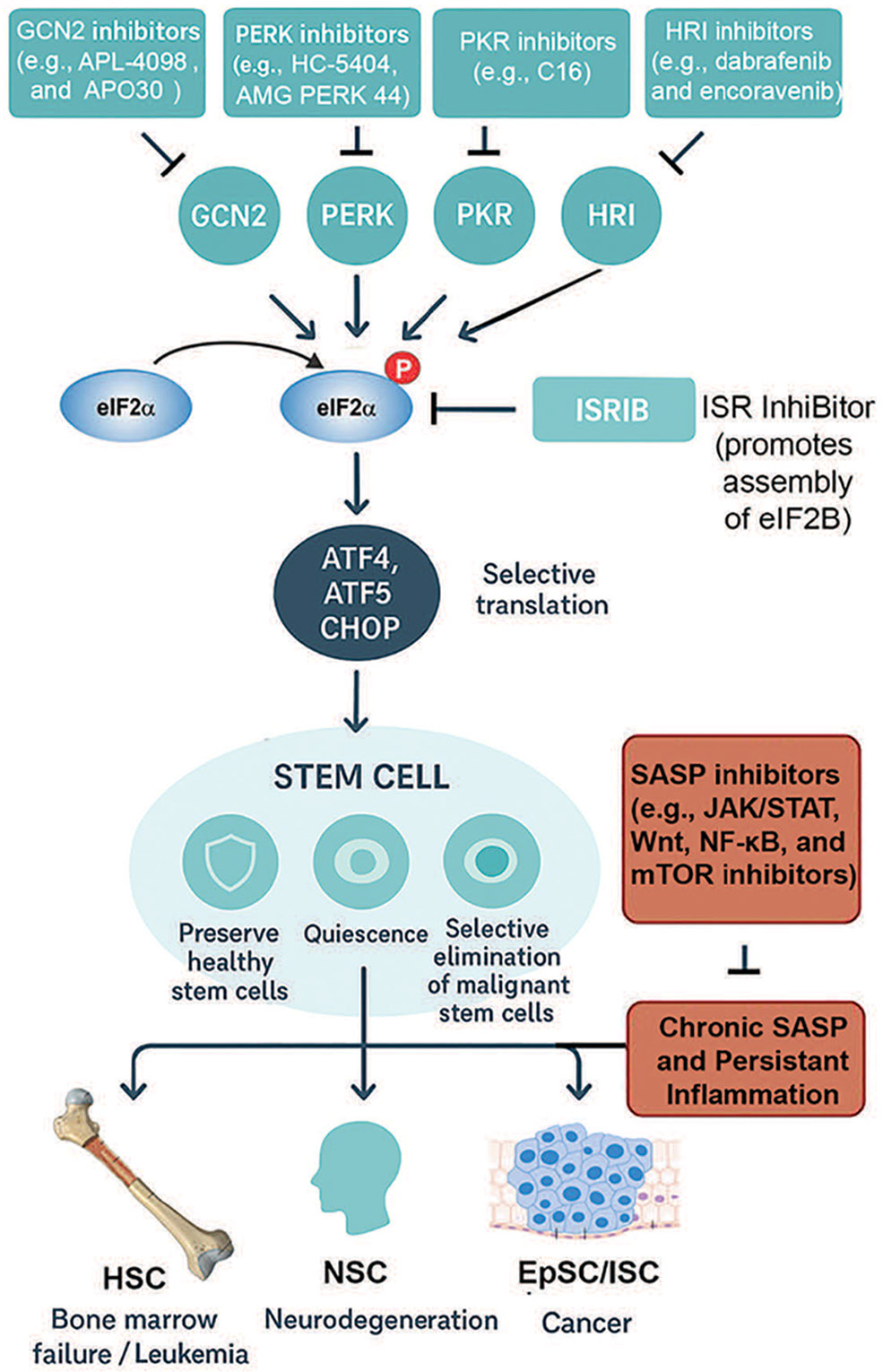
ISR outcomes and therapeutic implications in stem cell biology. The integrated stress response (ISR) pathway is shown with its four major stress-sensing kinases: PERK (endoplasmic reticulum stress), GCN2 (amino acid deprivation), PKR (viral dsRNA), and HRI (oxidative stress/heme depletion). These converge on phosphorylation of eIF2α, attenuating global protein synthesis while enabling ATF4-driven transcription. Pharmacological intervention points are highlighted: PERK inhibitors (e.g., GSK2606414, AMG44) block maladaptive ER stress; GCN2 modulators (e.g., tryptophan mimetics) tune amino acid–responsive signaling; HRI activators augment protective antioxidant programs; ISRIB rescues translation by antagonizing phospho-eIF2α effects at eIF2B. Disease contexts are illustrated as modules branching from stem cell populations: hematopoietic stem cells (HSCs; bone marrow failure, leukemia stem cells), neural stem cells (NSCs; neurodegeneration, repair), and epithelial stem cells (epidermal/intestinal; regeneration, cancer). Therapeutic goals are represented with icons: a shield symbolizes preservation of healthy stem cells, while a target indicates selective elimination of malignant or senescent cells. This schematic emphasizes how ISR and SASP modulation can be tailored to context, either protecting regenerative potential or suppressing pathological outcomes. Abbreviations: integrated stress response (ISR); senescence-associated secretory phenotype (SASP); eukaryotic initiation factor 2 alpha (eIF2α), Protein Kinase RNA-Like ER Kinase (PERK), General control nonderepressible 2 (GCN2), protein kinase R (PKR), heme-regulated inhibitor (HRI), activating transcription factor 4,5 (ATF4,5), C/EBP Homologous Protein (CHOP), hematopoetic stem cell (HSC), neural stem cell (NSC), intestinal stem cell (ISC), epithelial stem cell (EpSC), ISR InhiBitor (ISRIB), Janus kinase (JAK), signal transducer and activator of transcription (STAT), Wingless-related integration site (Wnt), Mechanistic target of rapamycin kinase (mTOR), nuclear factor kappa-light-chain-enhancer of activated B cells (NF-κB). ChatGPT5 was used to make early versions of the figures, which were improved with Photoshop

The intersection between the integrated stress response (ISR) and the senescence-associated secretory phenotype (SASP) provides a promising but complex therapeutic axis [11,73]. Both pathways serve as adaptive mechanisms: the ISR preserves stem cell integrity by buffering proteotoxic and metabolic stress, whereas the SASP coordinates tissue-level responses by releasing pro-inflammatory and remodeling factors [11,73]. However, when chronically engaged, their crosstalk can amplify dysfunction—driving bone marrow failure, neurodegeneration, and tumorigenesis (Fig. 3; Table 2) [11,73]. Thus, interventions must be designed to selectively restore beneficial signaling while suppressing maladaptive persistence.

One approach involves pharmacologic tuning of the ISR. Small-molecule inhibitors of eIF2α kinases (e.g., ISRIB analogs) can restore global translation and attenuate excessive ATF4-driven pro-apoptotic programs [4,38], thereby protecting hematopoietic and neural stem cell pools under chronic stress [4]. Conversely, transient ISR activation may be desirable in cancer, where sustained protein synthesis in malignant stem cells supports growth [80,81]. Here, PERK activators or modulators of amino acid-sensing pathways (GCN2 agonists) could shift damaged cells toward apoptosis rather than repair [2,75].

A complementary strategy is direct SASP modulation. Senolytics (e.g., dasatinib, navitoclax) eliminate senescent cells, reducing the inflammatory burden that exacerbates stem cell exhaustion [82-84]. Senomorphics, such as JAK inhibitors or mTOR modulators, instead dampen SASP factor production without killing senescent cells, preserving their initial tumor-suppressive arrest [83,85]. Notably, ISR–SASP crosstalk creates opportunities for synergy: ISR attenuation may reduce the persistence of DNA damage signaling that fuels SASP [4], while SASP suppression alleviates the pro-inflammatory milieu that chronically re-engages ISR pathways [6,86].

Context-specific modulation is critical. In regenerative medicine, transient ISR enhancement coupled with senomorphic therapy may preserve stem cell quiescence and prevent fibrosis during tissue repair [87]. In cancer therapy, the inverse may apply: ISR activators can drive tumor stem cells toward death, while senolytics remove SASP-amplified niches that support relapse [88]. Similarly, in neurodegenerative disease, balancing ISR inhibitors with SASP dampening could mitigate proteostasis collapse and chronic inflammation [89].

Ultimately, the therapeutic value of targeting ISR–SASP crosstalk lies in its dual-level control: intracellular stress adaptation and extracellular niche regulation. Precision in timing, dosage, and tissue context will determine whether interventions reinforce resilience or inadvertently accelerate decline.

## 4 Conclusion

The ISR and SASP are deeply integrated pathways that together orchestrate stem cell responses across development, regeneration, aging, and disease. The ISR acts as a master regulator of translation and survival, balancing adaptation and apoptosis to safeguard stem cell pools [1,2]. The SASP, long viewed as a pathological driver of aging, is now recognized as a dynamic program with critical roles in embryogenesis and wound healing [3]. Their convergence is most evident in senescent cells, where ISR signaling modulates SASP composition, amplifying inflammatory outputs with far-reaching consequences for tissue microenvironments and stem cell function [26,27].

Therapeutically, the ISR–SASP axis offers an attractive target for modulating stem cell fate in regenerative medicine, mitigating age-related decline, and controlling cancer progression [6,97,98]. Yet, interventions must be carefully tuned to distinguish between transient, beneficial roles and chronic, deleterious effects [6,97,98]. This review highlights the need for integrated frameworks that connect developmental biology with aging research, emphasizing that the ISR–SASP interplay represents both a biological constraint and an opportunity for therapeutic innovation.

## 5 Future Studies and Caveats

Future studies must dissect the temporal dynamics of ISR–SASP interactions at single-cell and spatial resolution, particularly within stem cell niches across development, adulthood, and aging [99]. Multi-omics approaches integrating translatomics, secretomics, and epigenomics will be crucial to map how ISR rewires SASP outputs [100]. Caveats include model-specific limitations: murine studies may not fully recapitulate human senescence programs, and *in vitro* stem cell assays often lack the complexity of *in vivo* microenvironments. Furthermore, interventions that blunt SASP or ISR could inadvertently disrupt essential developmental or regenerative functions. Addressing these challenges will be key to translating ISR–SASP biology into safe and effective therapies.

## Figures and Tables

**Table 1:** ISR and SASP in Stem Cell Fate Decisions

Example	Description	Reference(s)
HSC quiescence under nutrient stress	ISR activation preserves hematopoietic stem cell quiescence through ATF4-driven transcriptional programs that buffer metabolic stress and prevent premature differentiation.	[4,14]
Epidermal stem cells under amino acid deprivation	ISR shifts lineage output toward epidermal differentiation while delaying hair follicle regeneration, illustrating tissue-specific tuning of stem cell fate.	[15,16]
Neural stem cells after hypoxia	ISR activation via eIF2α phosphorylation directs NSCs toward differentiation or apoptosis depending on stress severity, acting as a developmental checkpoint.	[17,18]
Mesenchymal stem cells under ER stress	ISR activation can bias MSCs toward osteogenic rather than adipogenic lineages, showing ISR as a driver of fate transitions.	[19,20]
Senescent fibroblasts release IL-6 and IL-8.	SASP factors reshape the local microenvironment, promoting neighboring stem cell proliferation and differentiation but also potentially driving exhaustion with chronic exposure.	[6,21]
Senescent epithelial cells influence intestinal stem cells	SASP-mediated Wnt pathway activation stimulates intestinal stem cell expansion, enhancing regeneration after injury but risking dysplasia.	[22,23]
Senescent astrocytes affect neural stem cells	SASP cytokines impair neurogenesis and bias neural stem cells toward gliogenesis, contributing to age-associated cognitive decline.	[24,25]
ISR + SASP synergy in the bone marrow niche	Stress-primed ISR in HSCs combined with SASP from senescent stromal cells alters lineage bias, favoring myeloid over lymphoid differentiation in aging.	[26,27]
ISR–SASP crosstalk in skeletal muscle stem cells	ISR activation during stress sensitizes MuSCs to SASP-derived IL-1 and TNF, impairing self-renewal and driving premature senescence.	[4,28]
Cancer stem cell niches under chronic stress.	Combined ISR activation and SASP exposure remodel stemness pathways, fostering resistance and survival in tumor microenvironments.	[29-31]

Note: Abbreviations: hematopoetic stem cells (HSCs); Interleukin-6 and -8 (IL-6 and IL-8); mesenchymal stem cells (MSCs); muscle stem cells (MuSCs); integrated stress response (ISR); senescence-associated secretory phenotype (SASP); eukaryotic initiation factor 2 alpha (eIF2α); activating transcription factor (ATF); neural stem cells (NSCs); endoplasmic reticulum (ER).

**Table 2:** Therapeutic Modulation of ISR–SASP Crosstalk

Context	Pathway to target	Proposed intervention	Expected effect	Representative references
Acute injury and wound healing	Transient ISR activation (PERK/ATF4)	Pharmacologic ISR activators (e.g., halofuginone, salubrinal)	Promote adaptive survival and tissue repair	[4,90]
Chronic inflammation/fibrosis	Persistent SASP (NF-κB, JAK/STAT)	NF-κB inhibitors, JAK inhibitors (e.g., ruxolitinib)	Reduce inflammatory SASP and prevent fibrotic remodeling	[53,91]
Cancer prevention (premalignant lesions)	Maladaptive ISR–SASP loop	ISR inhibitors (PERK inhibitors, ISRIB) combined with SASP modulation	Limit tumor-promoting SASP while restoring normal stem cell fate	[92,93]
Aging-related stem cell exhaustion	Mixed ISR/SASP states	Senolytics (e.g., dasatinib + quercetin) and ISR modulators (ISRIB)	Remove senescent cells, rebalance stress adaptation in stem cell niches	[82,94]
Regenerative medicine (stem cell transplantation, reprogramming)	ISR-mediated quiescence	Fine-tuned ISR modulation (temporary activation, then inhibition)	Enhance stem cell survival during transplantation, improve reprogramming efficiency	[95,96]

Note: Abbreviations: Protein Kinase RNA-Like ER Kinase (PERK); nuclear factor kappa-light-chain-enhancer of activated B cells (NF-κB); Janus kinase (JAK); signal transducer and activator of transcription (STAT); ISR Inhibitor (ISRIB).

## Abbreviations

The following abbreviations are used in this manuscript

ISR: Integrated stress response
SASP: Stress-activated secretory pathway
ATF4: Activating transcription factor 4
eIF2α: Eukaryotic initiation factor 2 alpha
PERK: Protein Kinase RNA-Like ER Kinase
GCN2: General control nonderepressible 2
PKR: Protein kinase R
HRI: Heme-regulated inhibitor
ATF4,5: Activating transcription factor 4,5
CHOP: C/EBP Homologous Protein
HSC: Hematopoietic stem cell
NSC: Neural stem cell
ISC: Intestinal stem cell
EpSC: Epithelial stem cell
ISRIB: ISR Inhibitor
JAK: Janus kinase
STAT: signal transducer and activator of transcription
Wnt: Wingless-related integration site
NF-κB: Nuclear factor kappa-light-chain-enhancer of activated B cells
p53: p53 kinase
C/EBPβ: CCAAT/enhancer-binding protein beta
